# Association of pain management and positive expectations with psychological distress and spiritual well‑being among terminally ill cancer patients admitted to a palliative care unit

**DOI:** 10.1186/s12912-023-01259-z

**Published:** 2023-04-04

**Authors:** Yilong Yang, Meng Cui, Xinxin Zhao, Simeng Wang, Yumei Wang, Xiaohe Wang

**Affiliations:** 1 Department of Health Policy and Management, School of Public Health, Hangzhou Normal University, Hangzhou, P.R. China; 2 Hospice Ward, Shengjing Hospital of China Medical University, Shenyang, P.R. China; 3 Institute for International Health Professions Education and Research, China Medical University, Shenyang, P.R. China

**Keywords:** Pain management, Depression, Spiritual well-being, Palliative care, Positive expectations

## Abstract

**Background:**

Although palliation of psycho-spiritual distress is of great importance in terminally ill cancer patients, there is a little information about screening patients who benefit from palliative care and identifying the cancer care targets. This study explored the relationship of pain management and positive expectations with depression, anxiety and spiritual well-being (SWB) in terminal cancer patients admitted to a palliative care unit.

**Methods:**

Eighty-four terminal cancer inpatients were recruited from the Hospice Ward, Shengjing Hospital of China Medical University. Optimism and general self-efficacy (GSE) were evaluated at admission. Patients completed self-report questionnaires on SWB, depression, anxiety and pain both on admission and one week later. The repeated designed analysis of variance was used to explore the correlates of depression, anxiety and SWB (meaning, peace, faith).

**Results:**

In our sample, only cancer pain diminished significantly one week later. For depression (*p* = 0.041) and faith (*p* = 0.013), there was a significant pain group (relieved vs. not relieved) × time interaction effect, such that those with satisfied pain control experienced the improved psycho-spiritual outcomes at 1 week. The relationship between positive expectations, peace and faith was also statistically significant, indicating that the improvement of peace or faith was significant in the low group of optimism and GSE.

**Conclusions:**

Our findings indicated that pain management lied at the center of depression and SWB, meaning that effective pain management may reduce depression, and improve SWB among terminal cancer patients. Moreover, positive expectations, especially for optimism, may be the new target for SWB-related intervention research. Palliative care nurse should require the identification of terminal cancer patients who may more benefit from short-term palliative care, and target them with effective cancer care.

## Background

Terminal cancer patients are confronted with a limited life expectancy, suffer severe refractory symptoms, and face the fear of disability and helplessness [1, 2], in whom there is a descent in the quality of life (QoL). WHO advocated QoL beyond the physical, psychological and social dimensions, that is the fourth dimension that actively explored in persons with serious illness: spiritual well-being (SWB) [3]. Terminal patients struggled with spiritual issues about meaning and purpose of life, interpersonal connectedness, and religious problems. Additionally, several studies have highlighted the spiritual care as a fundamental component of palliative care (PC) [4, 5]. PC focuses on symptom control and psychosocial support in order to improve QoL for terminally ill patients, with subsequent changes to psychological and spiritual experience [6]. However, given the immaturity of palliative medicine, psycho-spiritual problems may be much serious in China, especially for SWB. For terminal cancer patients receiving PC, their fundamental purpose is to control physical symptoms rather than meet spiritual needs within short-term hospital stays [2]. Addressing spiritual issues also has not been a priority among nurses who carry out cancer treatment [7], leading to limited research about SWB in the PC practice. Moreover, none that we are aware of have profiled SWB change in short-term palliative setting.

SWB has been viewed as a multidimensional construct composed of 3 components: meaning, peace, and faith [8]. Meaning indicates the cognitive aspect of SWB and helps maintain a sense of meaning and purpose in life. Peace refers to the affective dimension of SWB and represents a sense of being reconciled to one’s adverse life circumstances. Faith is a sense of comfort or strength one derives from one’s spiritual beliefs as a foundation for understanding the world [9]. Although SWB is recognized as an indication of an individual’s QoL in the spiritual dimension, spiritual needs were rarely documented when terminally ill patients were referred to specialist PC services [10]. In addition, while many studies have demonstrated the prevalence of depression and anxiety in terminally ill cancer patients by self-report and clinical diagnosis [11–13], psychological distress tended to be underrecognized and undertreated in cancer patients [14]. Thus, in order to better improve QoL of terminal cancer patients receiving short-term PC, it is important to consider not only longitudinal changes in psychological distress and SWB but also the relevant factors of the longitudinal changes.

Terminal cancer patients often experience suffering of the whole person. According to the Biopsychosocial-Spiritual Model [15], it is critical to use a bio-cognitive-emotional approach to better understand what factors are associated with change in psychological distress and SWB. Pain is probably the most common and distressing symptom in the terminal phase of cancer [10, 16], resulting in the particularly high priority of pain management (PM) around the research and practice of PC. Our meta-analysis showed that PC was largely effective for relieving cancer pain [17]. Due to multi-factorial adverse impacts of cancer pain, the beneficial effects of PM should consider not only biomedical factors but also patients’ psychosocial and spiritual distress [18]. Lee et al. found that pain relief was associated with improved depression in advanced cancer patients receiving PC [19]. Lower level of pain severity and interference was related to higher SWB based on cross-sectional data [20]. In theory, pain is a complex subjective experience associated with bio-psycho-spiritual components. At the terminal stage of cancer, pain control can play a central role in managing a patient’s suffering, indicating that PM may influence psychological and spiritual issues. In practice, although terminally ill cancer patients experience both bio-psycho-spiritual sufferings, PC is mostly aimed at relieving pain using pharmacological strategies [17, 18]. As a result, our research question reads: to what extent has PM beneficial effects on psychological distress and SWB in the palliative setting.

The ability of health care providers to reduce sufferings requires the identification of patients who may more benefit from PC, and target them with effective interventions. Our cross-sectional studies indicated the beneficial effects of positive expectations (i.e., optimism and general self-efficacy, GSE) in cancer patients [21, 22], which both are generalized expectancies focused on desired outcomes and personal goal-achieving abilities. Optimism is defined as a relatively stable tendency to expect that good rather than bad things will happen, and positive outcome expectation will cause the continuous efforts in achieving the desired goal [23]. GSE, as a derivative construct of self-efficacy, refers to a relatively stable belief of personal competence to deal effectively with a variety of stressful situations [24]. Optimism and GSE were associated with lower psychological distress among cancer patients [11, 21, 22, 25, 26]. On the other hand, although positive expectations and SWB lies at the very center of patients with adjustment to cancer, not much was known regarding the association between optimism, GSE and SWB. A recent cross-sectional study found that SWB correlated positively with optimism in cancer patients initiating chemotherapy [25]. In a word, a perspective of positive expectations may yield a variety of psychological and spiritual benefits in cancer patients [11, 21, 22, 25, 26].

Given the importance of multi-factorial QoL in cancer and the dearth of research on longitudinal outcomes associated with PM and positive expectations, a greater understanding of psychological distress and SWB is warranted to help tailor the intervention of short-term PC. Given shifting trends of medical model in China, psycho-spiritual issues have attracted more and more attention in the field of palliative medicine. The current study adopted a prospective design to explore the relationship of PM and positive expectations with depression, anxiety and SWB in terminally ill cancer patients during the first week of admission to a PC unit. The 1-week period was chosen because the length of stay normally cannot exceed 2 weeks in the Chinese 3-Grade A hospitals.

## Methods

### Study design and participants

The prospective study was conducted of consecutive inpatients admitted to Shengjing Hospital of China Medical University between July 2019 and October 2020. Patients were recruited at the Hospice Ward as part of a larger study on transitional PC in terminal cancer patients. Eligible patients were at least 18 years of age and had a confirmed diagnosis of solid cancer. Other eligibility criteria were that patients had a life expectancy < 6 months at the time of admission and provided responses to study instruments with clear consciousness. Exclusion criteria were that patients had (1) concomitant psychiatric disorders, (2) psychotropic drug usage within 7 days before admission, (3) drug or alcohol dependence, and (4) HIV-positive. Eligible patients were identified through chart review and discussion with physicians.

### Procedures

All patients provided informed written consent prior to participation. Demographic information and medical history were obtained by research assistant through medical record review. Participants were asked to complete the psychometric instruments (i.e., SWB, depression, anxiety and pain) both on admission and one week after admission. About the interview schedule in detail, given the declining health and functions, patients decided themselves when they were available for interview, and thus our sample in stable condition finished the questionnaires. Because optimism and GSE are dispositional traits, participants completed the related scales at the time of admission. Based on each collection of valid questionnaires, enteral nutritional powder (ENSURE, 400 g per can) was given to patients as material incentives. The study was approved by the Committee on Human Experimentation of China Medical University (reference number: 720,042,321,006,479) and complied with the 1964 Helsinki Declaration and its later amendments.

### Implementation of PC

Patients were mainly referred to the Hospice Ward by their medical, surgical, or radiation oncologists. They were under active care focused on physical symptoms, provided by a medical team of physicians and nurses. PM mainly included pharmacological strategies (analgesic and adjuvant drugs), which were endorsed and promoted by WHO in the now-famous ‘analgesic ladder’ for managing cancer pain properly. Patients in this ward are usually seen over an average of one to two visits per day by a board-certified physician with a focus on pain and non-pain symptom management and nutrition support. PC nurses mainly provide routine cancer care. Due to staffing issues, fewer social workers and psychologists consult with patients as needed.

### Measures

#### Demographics and clinical variables

Demographic variables including age, gender, marital status, and education were collected at admission. Cancer type, diagnosis disclose, first or subsequent visit, Karnofsky Performance Scale (KPS) and Barthel Index were determined by medical chart review.

#### SWB

The Functional Assessment of Chronic Illness Therapy-Spiritual Well-being (FACIT-Sp) consists of 12 items measuring SWB with 3 empirically validated subscales of 4 items: meaning, peace, and faith [8]. Participants rated on a five-point Likert-type scale (0 = “not at all” to 4 = “very much”). In our study, the 3 separate subscales were used. Subscale scores range from 0 to 16, with higher scores reflecting higher levels of the three domains of SWB. Cronbach’s alphas for the three subscales were 0.743 (meaning), 0.633 (peace), and 0.916 (faith).

### Depression and anxiety

The HADS is a 14-item measure of depressive and anxiety symptoms [27]. HADS consists of two subscales (anxiety and depression), with 7 items each rated on a 4-point Likert scale (0 = “not at all” to 3 = “very much indeed”). Higher scores on both subscales indicate more severe symptoms of depression and anxiety. Cronbach’s alphas for the depression and anxiety subscales were 0.876 and 0.869, respectively.

### Pain severity

Cancer pain was assessed by the Numerical Rating Scales (NRS), which is one of the frequently used tools for assessing pain severity [2, 17]. Patients were asked to verbally rate their pain on a scale from 0 to 10, with 0 equals no pain and 10 equals worst possible pain. The 11-point NRS is easy to administer verbally among terminal cancer patients admitted to the palliative setting.

### Optimism

The Life Orientation Scale-Revised (LOT-R) is a 10-item measure of dispositional optimism, which comprises 6 items (3 positively worded and 3 negatively worded items) and 4 filler items [23]. Each item is rated on a 5-point Likert scale, ranging from 1 (strongly disagree) to 5 (strongly agree). The total of the 3 positively worded items was calculated as the indicator of optimism in our study [2, 22], and higher scores suggested higher level of optimism. Cronbach’s alpha for this study was 0.774.

#### GSE

The General Self-Efficacy Scale (GSES) was employed to assess GSE [21, 22], which consists of 10 items rated on a 4-point Likert scale (1 = “not at all true” to 4 = “exactly true”). The total score ranges from 10 to 40 scores, and higher score indicates higher level of GSE. Cronbach’s alpha for the GSES was 0.929.

### Statistical analysis

Descriptive statistics were used to summarize participants characteristics and study variables. To dichotomize our sample due to degrees of optimism and GSE, we used the median scores of the distributions as the cut-off criteria [26]. Based on the method of Lee et al. [19], patients were assigned to one of two groups according to whether their NRS scores were lower one week after admission or not. The patients whose NRS scores indicated one week after later were lower than those on admission were assigned to the relieved group; the others were assigned to the not relieved group. Independent samples *t*-test evaluated difference in pain, depression, anxiety and FACIT-Sp subscales between the two groups of dichotomized variables at each assessment time point. Paired samples *t*-test assessed changes in pain, depression, anxiety and FACIT-Sp subscales between the two assessment time points for each group. The repeated designed analysis of variance (ANOVA) with one between-subject factor “group” and one within-subject factor “time” were used to explore the association of PM and positive expectations with depression, anxiety and SWB. Unstandardized simple slopes were probed and plotted to visualize the interaction term of group and time. The Statistical Package for the Social Sciences (SPSS, version 18.0) was used to perform the statistical analyses, with two-tailed probability value of < 0.05 considered to be statistically significant.

## Results

### Patient characteristics

**Fig. 1:**
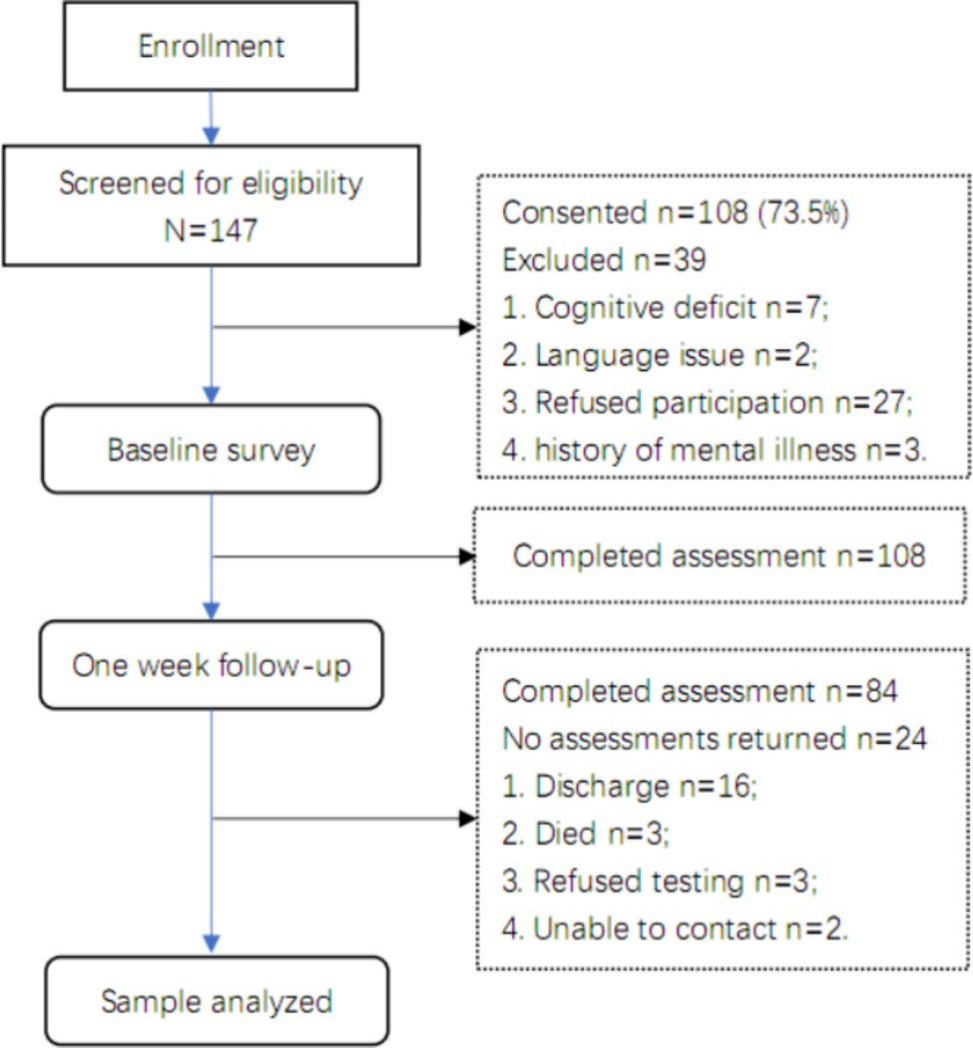
Flow diagram through the study

As shown in Fig. 1, there were 108 eligible patients at baseline. Of these patients, 84 completed assessments at 1-week follow-up. The main reason for missing data was that patients were discharged from hospice ward at follow-up (attrition rate = 22.2%). As shown in Table 1, the mean age of patients was 66.29 years. Patients were female (57.1%), married/living with a partner (73.8%), and middle school educated (51.2%). The most common primary cancer sites included gastrointestinal (42.9%) and lung (25%). Most patients were aware of cancer diagnosis (63.1%), and were the first time to the Hospice Ward (77.4%). For KPS scores, the mean (SD) was 46.39 ± 8.91. Additionally, one week after admission, 40 (47.6%) patients reported an improvement in pain (relieved group).

**Table 1:** Demographic and clinical characteristics of participants (N = 84)

Variable	N(%)	Mean (SD)	Median(min-max)	95% CL
**Demographic information**				
Age		66.29(12.99)	65.5(32–91)	63.48–69.12
Gender				
Male	36(42.9)			
Female	48(57.1)			
Marital status				
Married/living with partner	62(73.8)			
Single/separated/widowed/ divorced	21(25)			
Education				
Middle school or below	43(51.2)			
High school	20(23.8)			
Junior college or above	20(23.8)			
**Clinical information**				
Cancer type				
Genitourinary	4(4.8)			
Gastrointestinal	36(42.9)			
Lung	21(25)			
Gynecological	7(8.3)			
Breast	8(9.5)			
Head/neck	2(2.4)			
Others	6(7.1)			
Aware of cancer diagnosis				
Yes	53(63.1)			
No	30(35.7)			
KPS score (range 0–100)		46.39 (8.91)	50 (30–70)	44.44–48.33
< 50	40 (47.6)			
≥ 50	43 (51.2)			
First time to PCU				
Yes	65(77.4)			
No	19(22.6)			
Barthel index		40.18(24.54)	40(0-100)	34.82–45.54

Variable containing missing information: marital status (1, 1.2%); education (1, 1.2%); aware of cancer diagnosis (1, 1.2%); KPS score (1, 1.2%); Barthel index (1, 1.2%)

Abbreviation: SD, standard deviation; KPS, Karnofsky Performance Scale; 95% CI: 95% confidence interval; PCU, palliative care unit

### Univariate analysis

Table 2 compared the assessments of repeated measures between the two groups of dichotomized variables at each assessment time point. NRS scores were significantly higher in the relieved group (*p* < 0.001) at admission. One week after admission, NRS scores were significantly lower in the relieved group (*p* = 0.032). Depression, anxiety and pain were significantly higher in the low optimism group, and the three subscales of FACIT-Sp were significantly higher in the high optimism group at admission. Similar results were observed one week later, except for peace (*p* = 0.112) and pain (*p* = 0.063). Additionally, the three subscales of FACIT-Sp were significantly higher in the high GSE group at admission. One week after admission, only meaning scores were significantly higher in the high GSE group (*p* = 0.036).

**Table 2:** Distributions of psychological distress, spiritual well-being and pain in different groups of pain management, optimism and general self-efficacy

Variable	Pain management,					Optimism					General self-efficacy				
	Relieved (N = 40)		Not relieved (N = 44)			High N = 42		Low N = 42			High N = 42		Low N = 42		
	Mean ± SD		Mean ± SD	t	*p*	Mean ± SD		Mean ± SD	t	*p*	Mean ± SD		Mean ± SD	t	*p*
At admission															
Depression	11.18 ± 5.43		9.94 ± 5.43	-1.043	0.3	7.59 ± 4.67		13.47 ± 4.49	5.881	< 0.001	9.48 ± 5.54		11.57 ± 5.18	1.785	0.078
Anxiety	11.03 ± 5.01		9.48 ± 4.84	-1.444	0.153	7.98 ± 4.56		12.45 ± 4.32	4.612	< 0.001	9.48 ± 5.42		10.95 ± 4.39	1.368	0.175
Meaning	9.41 ± 3.27		9.77 ± 3.99	0.456	0.649	11.78 ± 2.89		7.41 ± 2.96	-6.845	< 0.001	10.49 ± 3.89		8.69 ± 3.19	-2.325	0.023
Peace	7.43 ± 3.06		8.25 ± 3.41	1.15	0.253	9.65 ± 2.98		6.07 ± 2.45	-6.012	< 0.001	8.82 ± 3.67		6.9 ± 2.47	-2.811	0.006
Faith	6.04 ± 4.24		7.59 ± 5.37	1.458	0.149	9.79 ± 4.49		3.9 ± 3.24	-6.893	< 0.001	8.85 ± 5.39		4.85 ± 3.36	-4.072	< 0.001
Pain	7.89 ± 1.86		5.39 ± 3.48	-4.138	< 0.001	5.56 ± 3.44		7.6 ± 2.28	3.197	0.002	6.04 ± 3.33		7.12 ± 2.74	1.632	0.107
One week after admission															
Depression	9.51 ± 5.31		10.36 ± 5.61	0.712	0.478	7.44 ± 4.77		12.48 ± 4.94	4.752	< 0.001	8.9 ± 5.72		11.01 ± 5.01	1.795	0.076
Anxiety	9.43 ± 4.31		9.5 ± 5.14	0.07	0.944	7.57 ± 4.21		11.36 ± 4.51	3.977	< 0.001	8.76 ± 4.83		10.17 ± 4.58	1.365	0.176
Meaning	9.45 ± 3.55		8.98 ± 3.92	-0.572	0.569	10.88 ± 3.28		7.53 ± 3.42	-4.586	< 0.001	10.05 ± 3.8		8.35 ± 3.51	-2.129	0.036
Peace	7.97 ± 2.62		7.86 ± 3.46	-0.164	0.87	8.45 ± 3.41		7.38 ± 2.63	-1.608	0.112	8.3 ± 3.55		7.52 ± 2.49	-1.169	0.246
Faith	7.61 ± 4.15		6.92 ± 4.92	-0.698	0.487	8.88 ± 4.47		5.61 ± 4.07	-3.51	0.001	8.17 ± 5.03		6.32 ± 3.87	-1.885	0.063
Pain	5.23 ± 2.37		6.55 ± 3.06	2.185	0.032	5.35 ± 2.76		6.49 ± 2.78	1.883	0.063	5.7 ± 3.04		6.14 ± 2.59	0.713	0.478

Abbreviation: SD, standard deviation

Table 3 assessed changes in pain, depression, anxiety and FACIT-Sp subscales between the two assessment time points for each group. At 1-weel follow-up, a significant improvement in depression/anxiety, faith and pain was indicated by the pain relieved group (*p* < 0.05), but the linear trend was not significant in the not relieved group. A significant decline in meaning and peace was observed in the high optimism group, and a significant improvement was reported by the low optimism group one week later. In addition, a significant increase in faith was reported by the low GSE group one week after admission (*p* = 0.022).

**Table 3:** Distributions of psychological distress, spiritual well-being and pain at different time points

Variable	At admission		One week after admission		
	Mean ± SD		Mean ± SD	t	*p*
Pain relieved (N = 40)					
Depression	11.18 ± 5.43		9.51 ± 5.31	2.045	0.048
Anxiety	11.03 ± 5.01		9.43 ± 4.31	2.257	0.03
Meaning	9.41 ± 3.27		9.45 ± 3.55	-0.13	0.897
Peace	7.43 ± 3.06		7.97 ± 2.62	-1.35	0.185
Faith	6.04 ± 4.24		7.61 ± 4.15	-2.376	0.022
Pain	7.89 ± 1.87		5.23 ± 2.37	8.751	< 0.001
Pain not relieved (N = 44)					
Depression	9.94 ± 5.43		10.36 ± 5.61	-0.693	0.492
Anxiety	9.48 ± 4.84		9.5 ± 5.14	-0.033	0.973
Meaning	9.77 ± 3.99		8.98 ± 3.92	1.752	0.087
Peace	8.25 ± 3.41		7.86 ± 3.46	0.77	0.445
Faith	7.59 ± 5.37		6.92 ± 4.92	1.127	0.266
Pain	5.39 ± 3.48		6.55 ± 3.06	-4.397	< 0.001
High optimism (N = 42)					
Depression	7.59 ± 4.67		7.44 ± 4.77	0.211	0.834
Anxiety	7.98 ± 4.56		7.57 ± 4.21	0.682	0.499
Meaning	11.78 ± 2.89		10.88 ± 3.28	2.179	0.035
Peace	9.65 ± 2.98		8.45 ± 3.41	2.711	0.01
Faith	9.79 ± 4.49		8.88 ± 4.47	1.384	0.174
Pain	5.56 ± 3.44		5.35 ± 2.76	0.493	0.625
Low optimism (N = 42)					
Depression	13.47 ± 4.49		12.48 ± 4.94	1.289	0.205
Anxiety	12.45 ± 4.32		11.36 ± 4.51	1.378	0.176
Meaning	7.41 ± 2.96		7.53 ± 3.42	-0.303	0.763
Peace	6.07 ± 2.45		7.38 ± 2.63	-3.255	0.002
Faith	3.9 ± 3.24		5.61 ± 4.07	-3.006	0.005
Pain	7.6 ± 2.29		6.49 ± 2.78	2.989	0.005
High general self-efficacy (N = 42)					
Depression	9.48 ± 5.54		8.9 ± 5.72	0.923	0.361
Anxiety	9.48 ± 5.42		8.76 ± 4.83	1.034	0.307
Meaning	10.49 ± 3.89		10.05 ± 3.8	1.054	0.298
Peace	8.82 ± 3.67		8.3 ± 3.55	0.991	0.327
Faith	8.85 ± 5.39		8.17 ± 5.03	1.061	0.295
Pain	6.04 ± 3.33		5.7 ± 3.04	0.914	0.366
Low general self-efficacy (N = 42)					
Depression	11.57 ± 5.18		11.01 ± 5.01	0.68	0.5
Anxiety	10.95 ± 4.39		10.17 ± 4.58	1.096	0.279
Meaning	8.69 ± 319		8.35 ± 3.51	0.85	0.4
Peace	6.9 ± 2.47		7.52 ± 2.49	-1.612	0.115
Faith	4.85 ± 3.36		6.32 ± 3.87	-2.382	0.022
Pain	7.12 ± 2.74		6.14 ± 2.59	2.248	0.03

Abbreviation: SD, standard deviation

### Multivariable analysis

We used a repeated designed ANOVA to evaluate the association of PM and positive expectations with depression, anxiety and SWB. Table 4 indicated that no main effect of time was detected for study variables. The main effect of optimism and GSE was observed for depression, anxiety and three subscales of FACIT-Sp (*p* < 0.05). For faith domain, there was a significant group × time interaction effect detected, indicating that the group effect of PM (*p* = 0.013), optimism (*p* = 0.003) and GSE (*p* = 0.018) on faith was dependent on time. Interaction of pain/optimism and time was significantly associated with depression (PM group, *p* = 0.041) or peace (optimism group, *p* < 0.001).

**Table 4:** Interaction of group and time on psychological distress and spiritual well-being (N = 84)

	Group			Time			Group×Time	
Variable	F	*p*		F	*p*		F	*p*
**Group: Pain management**								
Depression	0.032	0.858		1.504	0.224		4.293	0.041
Anxiety	0.624	0.432		2.587	0.112		2.738	0.102
Meaning	0.005	0.946		1.68	0.199		2.105	0.151
Peace	0.334	0.565		0.05	0.823		2.017	0.159
Faith	0.212	0.646		1.033	0.312		6.391	0.013
**Group: Optimism**								
Depression	37.377	< 0.001		1.221	0.272		0.68	0.412
Anxiety	25.23	< 0.001		2.283	0.135		0.478	0.491
Meaning	38.263	< 0.001		1.892	0.173		3.212	0.077
Peace	17.515	< 0.001		0.029	0.865		17.589	< 0.001
Faith	34.437	< 0.001		0.829	0.365		9.059	0.003
**Group: General self-efficacy**								
Depression	3.977	0.049		1.211	0.274		0	0.986
Anxiety	2.407	0.125		2.27	0.136		0.005	0.945
Meaning	5.734	0.019		1.822	0.181		0.033	0.855
Peace	5.183	0.025		0.025	0.875		3.077	0.083
Faith	11.169	0.001		0.8	0.374		5.849	0.018

**Fig. 2:**
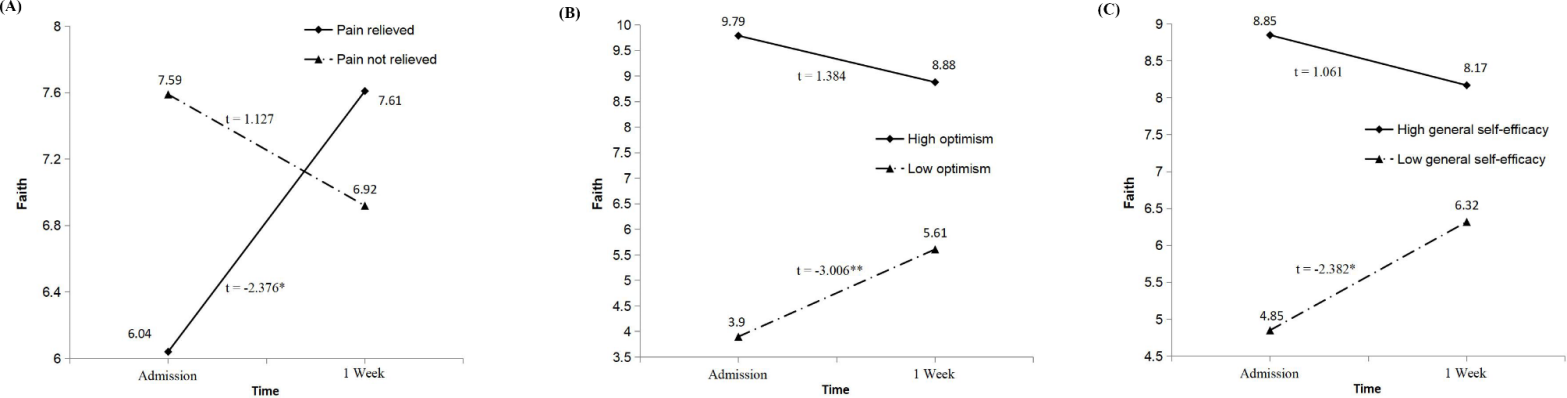
Interaction of pain management (A), optimism (B), general self-efficacy (C) and time on faith **Note**: Paired samples *t*-test was used to compare faith between the two assessment time points for each group Abbreviation: * *p* < 0.05; ** *p* < 0.01

As shown in Fig. 2A, faith in the pain relieved group was significantly increased one week later, but the linear change was not significant in the not relieved group. The improvement of faith was significant in the low group of optimism and GSE, while faith remained relatively stable over time in the group of high optimism (*p* = 0.174) and GSE (*p* = 0.295) (see Fig. 2B C).

**Fig. 3:**
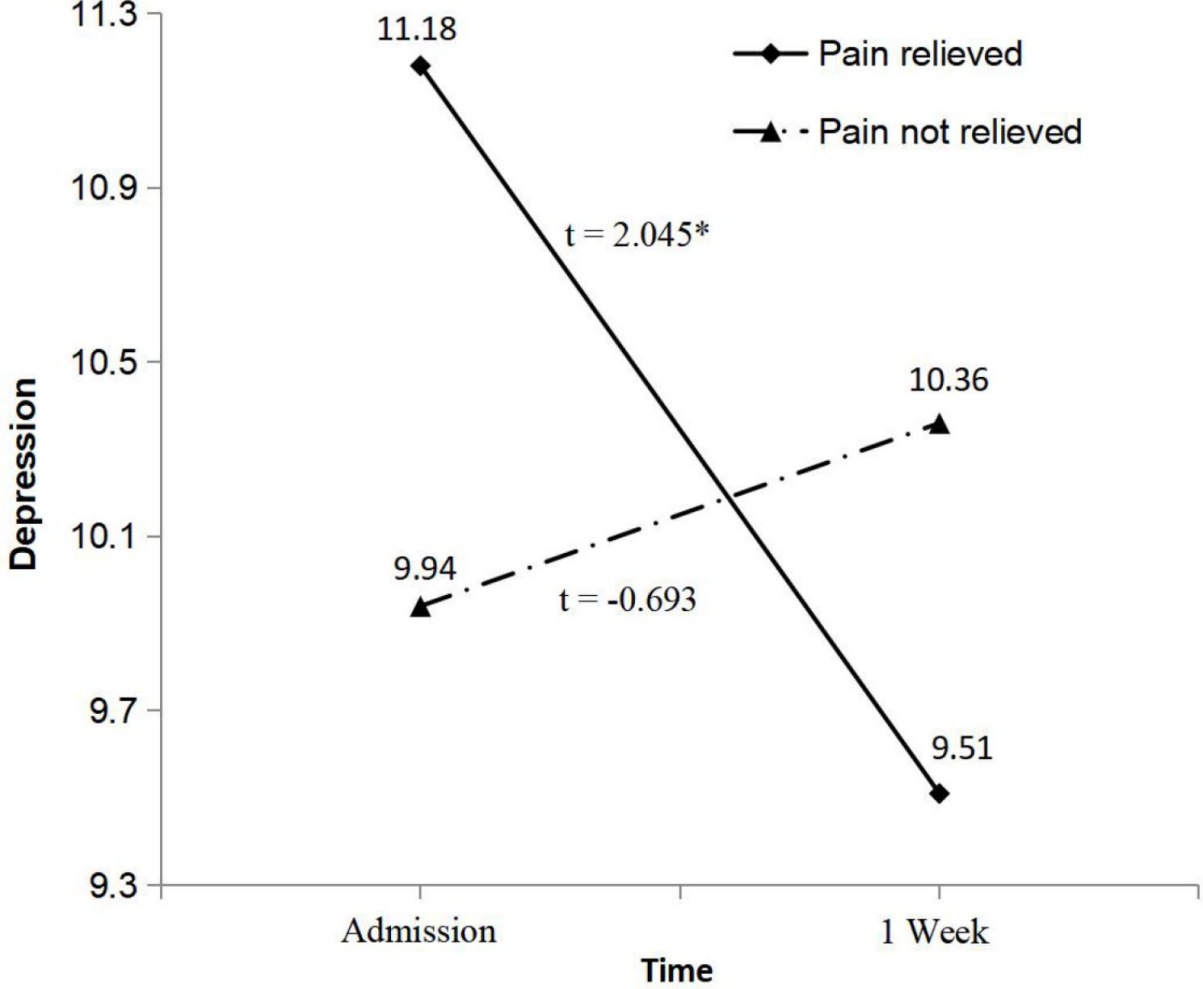
Interaction of pain management and time on depression **Note**: Paired samples *t*-test was used to compare depression between the two assessment time points for pain management group Abbreviation: * *p* < 0.05

**Fig. 4:**
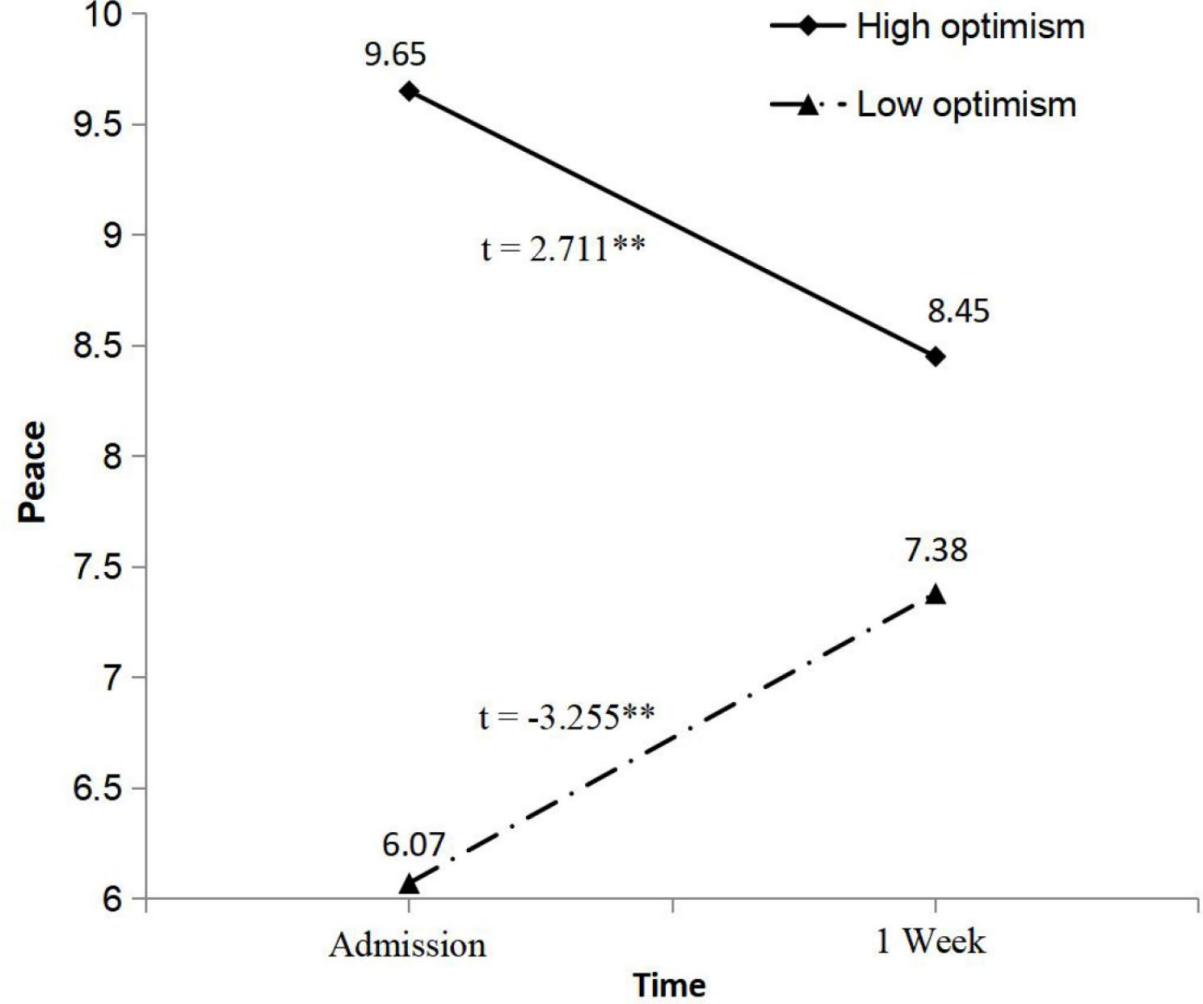
Interaction of optimism and time on peace **Note**: Paired samples *t*-test was used to compare depression between the two assessment time points for optimism group Abbreviation: ** *p* < 0.01

In the pain relieved group, depression was significantly reduced one week after admission, while the change of depression was not significant in the not relieved group (see Fig. 3). Figure 4 showed that one week after admission, a significant improvement in peace was revealed by the low optimism group (*p* = 0.002), while patients reported a significant decline in peace in the high group (*p* = 0.01).

## Discussion

Our findings should be interpreted with caution given the restrictions during COVID-19. Several studies found that the limited interaction between patients and family members is one of major challenges in Chinese PC units during COVID-19 [28, 29]. Because of closed-off management and strict visitation system, access to the Hospice Ward is restricted for family members of our sample, which leads to the absence of families’ company and the long-term social distancing. Terminal cancer patients under this situation may have psychosocial and spiritual issues, and so the restrictions during COVID-19 could be of major influence on feelings of depression and SWB.

Cancer pain diminished significantly in our sample receiving PC for one week (t = 2.303, *p* = 0.024), but depression, anxiety and SWB did not change significantly during 1-week period (results of t test not shown). Our previous meta-analysis found that PC was largely effective to relieve pain in Chinese adults with cancer [17]. Research around PM is also of particularly high priority in mainland China [30]. By contrast, addressing spiritual issues has not been a priority in PC nurse where the word ‘spiritual’ is not widely accepted or used in China [7, 30]. More importantly, health care providers lack relevant knowledge and teaching about how to deal with psycho-spiritual problems in Chinese palliative settings. This study highlighted the necessary preparations for PC nurses to manage psycho-spiritual issues in Chinese terminal cancer patients.

For depression scores, there was a statistically significant PM group × time interaction effect. Lee et al. found the similar relationship between depression and PM in advanced cancer patients [19]. Recent systematic review reported that cancer pain was identified as a significant predictor for depression [31]. Furthermore, clustering of cancer pain and depression suggested a common underlying etiology resulted from inflammatory cytokines, such as elevated interleukin-6 [32]. The specific type of PM had significant reductions in depressed level of consciousness supported by a randomized clinical trial [33], demonstrating that interventions focused on PM may lead to improvement in mood. Thus, cancer pain was significantly improved by short-term pharmacological strategy (e.g., opioid analgesics), which may ameliorate depressive symptoms of our sample.

The most important findings were that the group effect of PM, optimism and GSE on faith was dependent on time, and the different linear trend of faith was indicated in the Fig. 2. There are several moderating factors we should take into consideration during short-term PC. Figure 2 A showed that PM affected the experience of faith. Faith refers to the feelings of comfort and strength derived from one’s spiritual beliefs, which could be considered as a foundation for understanding the world [7–9]. Individuals find great comfort and strength in their faith while facing uncontrollable distress, which highlights the role of faith in cancer adjustment to some extent [34]. Empirical studies revealed that higher SWB was associated with lower pain severity and intensity among patients with cancer and spinal cord injury [20, 35]. Therefore, under the effective control of cancer pain, our sample may draw greater comfort and strength from their spiritual beliefs in coping with terminal cancer.

For each group of positive expectations, similar linear trend was observed in the faith (see Fig. 2B C). Possible mechanism linking positive expectations and faith might be the effect of PM. At admission, faith was significantly lower in the low group of positive expectations, which was in line with cross-sectional studies reporting the positive association between positive expectations and SWB [25, 36]. One week after admission, pain relief was observed in the low group of positive expectations. Given the PM-faith association discussed above (see Fig. 2A), we speculated that PM lies at the very center of psycho-spiritual issues, which is a modifiable variable in determining whether our sample with low positive expectations would experience higher faith over time.

We also found the relationship of optimism with peace. Peace can be experienced when an individual has reached acceptance of a particular life challenge and pursues meaningful goals [9]. Considered as an outcome expectation [21–23], optimism may lead to higher peace at admission by enabling patients to expect positive health-related outcomes and maintain the efforts to attain the desired goals. However, desired goals should be realistic and achievable in the terminal stage. As shown in Fig. 4, possible explanation may be that physical deterioration gave an increased sense of doubt, inactivity, and uncertainty, and optimistic patients with favorable expectations may not still experience high peace level during 1-week follow-up. In contrast, given the lower peace at admission, patients with low optimism may be more likely to improve peace during short-term PC.

## Clinical implications

A better understanding of depression and SWB may be very important for effective cancer care in the palliative settings. Psycho-spiritual care should be an essential element of short-term PC for terminally ill cancer patients. For instance, in addition to pain relief, PC nurses should recognize the improvement in depression and SWB underlying effective PM. Additionally, amelioration in cancer pain may also be associated with improved SWB in the low group of positive expectations. On the other hand, optimism may be the new target for spirituality-related intervention research. PC professionals should pay more attention to providing targeted cancer care for patients with low optimism, which may be beneficial in increasing peace during short-term PC.

## Limitations

The present findings should be contemplated within the context of the study’s limitations. First, observational design precluded drawing conclusions regarding causality. For instance, PM was associated with over-time changes in depression, but the opposite conclusion that depression predicted subsequent effect of pain control may be also true. The reciprocal pain-depression relationship is evidenced by their clinical presentations and anatomic regions in the brain [37]. Second, because of no control group in our study, we cannot know how peace and faith change over time for each group of positive expectations without PM. Third, the strict quarantine measures resulted in a small simple size during COVID-19 pandemic. Our findings should be interpreted with caution given the sample size and the convenience sample. Fourth, we may not capture the full extent of changes in cancer pain, psychological distress and SWB for 1-week observation period. Future research should explore changes during long-term PC. Finally, NRS is a single-dimensional numerical rating scale, limiting the more comprehensive evaluation related to the components of pain.

## Conclusions

The present study furthered the understanding of depression and SWB in terminally ill cancer patients receiving short-term PC. PM was associated with an improvement of depression and faith. There was a significant relationship between positive expectations and faith. Moreover, interaction of optimism × time was significantly associated with peace. More effective PM may improve depression and SWB among terminal cancer patients. For peace domain of SWB, our findings have implications for screening patients with low optimism who may get more benefit from the short-term PC.

## Data Availability

The datasets used and/or analyzed during the current study available from the corresponding author on reasonable request.

## List of abbreviations

ANOVA: Analysis of Variance
COVID-19: Coronavirus Disease 2019
FACIT-Sp: Functional Assessment of Chronic Illness Therapy-Spiritual Well-being
GSE: General self-efficacy
GSES: General Self-Efficacy Scale
HADS: Hospital Anxiety and Depression Scale
LOT-R: Life Orientation Scale-Revised
NRS: Numerical Rating Scales
PC: Palliative care
PM: Pain management
QoL: Quality of life
SWB: Spiritual well-being
